# Effect of edaravone on pregnant mice and their developing fetuses subjected to placental ischemia

**DOI:** 10.1186/s12958-021-00707-2

**Published:** 2021-02-06

**Authors:** Marwa Atallah, Toru Yamashita, Koji Abe

**Affiliations:** 1 Department of Neurology, Okayama University Graduate School of Medicine, Dentistry and Pharmaceutical Sciences, 2-5-1 Shikata-cho, Okayama, 700-8558 Japan; 2 Vertebrates Comparative Anatomy and Embryology, Zoology Department, Faculty of Science, Menoufia University, Shebin El-Koom, Egypt

**Keywords:** Preeclampsia, RUPP, Edaravone, Placental ischemia, Endoskeleton, Fetal growth restriction

## Abstract

Growing evidence indicates that reduced uterine perfusion pressure (RUPP) triggers the cascade of events leading to preeclampsia. Edaravone is a powerful free radical scavenger used for the treatment of ischemia/reperfusion diseases due to its anti-oxidative stress and anti-inflammatory properties. Here we investigate the effect of edaravone (3 mg/kg) on different maternal and fetal outcomes of RUPP-induced placental ischemia mice model. RUPP surgery was performed on gestation day (GD) 13 followed by edaravone injection from GD14 to GD18, sacrifice day. The results showed that edaravone injection significantly decreased the maternal blood pressure (113.2 ± 2.3 mmHg) compared with RUPP group (131.5 ± 1.9 mmHg). Edaravone increased fetal survival rate (75.4%) compared with RUPP group (54.4%), increased fetal length, weights, and feto-placental ratio (7.2 and 5.7 for RUPP and RUPP-Edaravone groups, respectively) compared with RUPP group. In addition, RUPP resulted in many fetal morphological abnormalities as well as severe delayed ossification, however edaravone decreased the morphological abnormalities and increased the ossification of the fetal endoskeleton. Edaravone improved the histopathological structure of the maternal kidney and heart as well as decreased the elevated blood urea and creatinine levels (31.5 ± 0.15 mg/dl (RUPP), 25.6 ± 0.1 mg/dl (RUPP+edaravone) for urea and 5.4 ± 0.1 mg/dl (RUPP), 3.5 ± 0.1 mg/dl (RUPP+edaravone) for creatinine) and decreased cleaved caspase-3 expression in the maternal kidney. In conclusion, this study demonstrated that our RUPP mice model recapitulated preeclampsia symptoms and edaravone injection ameliorated most of these abnormalities suggesting its effectiveness and potential application in preeclampsia treatment regimes.

**Supplementary Information:**

The online version contains supplementary material available at 10.1186/s12958-021-00707-2.

## Background

Reduced placental blood flow leads to placental ischemia, an initiating event in the pathophysiology of preeclampsia [11]. The pathogenesis of preeclampsia is related to trophoblast invasion suppression leading to incomplete spiral artery remodeling and decreased blood flow to the placenta (Stage 1 of preeclampsia) which results in placental hypoxia/ischemia and poor placental growth (stage 2) [15, 58]. All these changes cause oxidative stress in placental villi [20], which contribute to maternal endothelial dysfunction, leading to hypertension, renal impairment, proteinuria, and perinatal death [30]. Intrauterine fetal growth restriction has been associated with preeclampsia which occurs due to the insufficient supply of oxygen and/or nutrients to the fetus [43]. Currently, there are no effective treatments for preeclampsia, except for the delivery of babies, however, premature delivery often causes severe complications in the neonates [50].

The reduced uterine perfusion pressure (RUPP) model is a well-characterized animal model of preeclampsia [11, 27, 32] which has been reported to manifest symptoms related to preeclampsia such as increased maternal blood pressure, increased serum sFlt-, proteinuria, endotheliosis and decreased fetal weight [17, 23]. Additionally, it has been reported that RUPP model is also a model of oxidative stress observed during preeclampsia [40]. Thus, the RUPP model is ideal to understand pathophysiological mechanisms underlying preeclampsia associated with placental ischemia [11].

Uteroplacental ischemia results in hypoxia due to reduced blood flow to the placenta and developing fetuses. As a result, the production of free radicals and ROS is increased, causing excessive apoptosis of trophoblast cells and endothelial dysfunction. Based on the fact that oxidative stress plays important role in the pathogenesis of preeclampsia [37], the authors discussed the preventive and therapeutic potential of some antioxidants, such as Vitamin E and C in preeclampsia. Moreover, Ushida et al. [50] reported that the administration of H2, a novel antioxidant, improved the placental ischemia-induced hypertension, angiogenic imbalance, and oxidative stress.

Edaravone (3-methyl-1-phenyl-2-pyrazolin-5-one, synonyms MCI-186) is a potent free radical scavenger [28] whose administration was found to improve ROS-induced tissue damage [47]. It has protective effects mainly against oxidative stress-induced neurodegenerative diseases [34], however, studies have demonstrated its effectiveness in other tissues [22, 24, 49, 56]. Moreover, Zhao et al. [58] demonstrated that edaravone decreased ROS production and sFlt-1 secretion in an in vitro model of placental hypoxia and suggested that edaravone can be used as a therapeutic target for the prevention of preeclampsia. Consequently, the aim of the present study was to investigate, for the first time, whether in vivo administration of edaravone could improve maternal and fetal pathophysiological characteristics related to preeclampsia using the RUPP mice model.

## Materials and methods

### Animals

All animal experiments were performed in compliance with a protocol approved by the Animal Committee of the Graduate School of Medicine and Dentistry, Okayama University (OKU-2019677). Healthy mature virgin females and fertile males of C57BL/6 N mice (22 ± 2 g, 8 ± 1 week old) were purchased from SLC, Japan (Shizuoka, Japan). The mice were housed in a temperature-regulated room (23–25 °C) with free access to food and water, under a 12 h light/12 h dark cycle.

### RUPP surgery as a model to induce placental ischemia

To induce placental ischemia, the reduced uterine perfusion pressure (RUPP) model was adopted with a modification from Janot et al. [25]. Mating (2 females/1 male) was achieved. The day at which vaginal plug was located has been considered as day zero of pregnancy. Gestation day (GD)13 was determined to be the RUPP surgery day and GD18 was determined as the end point for experimentation.

**Fig. 1:**
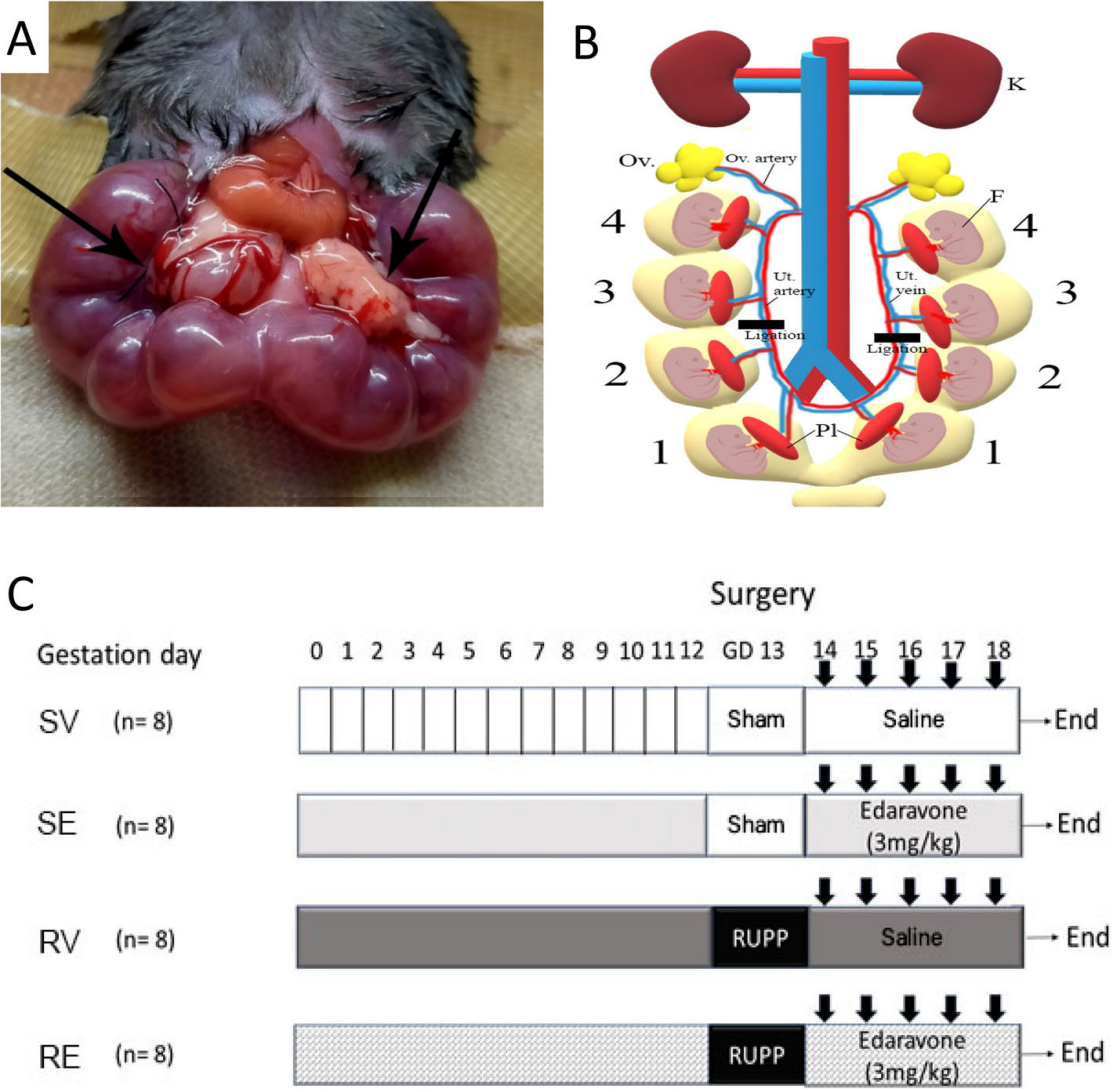
**a** Ligation of the two uterine vessels was performed in pregnant mice of experimental groups around the middle of each uterine horn. **b** Schematic description of the surgery. **c** Experimental groups included SV, sham surgery plus i.v. injection of 0.3 ml saline. SE group, sham surgery plus i.v. injection with 0.3 ml edaravone (3 mg/kg). RV group surgery with ligation of both uterine vessels plus i.v. injection of 0.3 ml saline. RE group surgery with ligation of both uterine vessels plus i.v. injection of 0.3 ml edaravone (3 mg/kg). All injections started at GD14 and ended at GD 18. F; fetus, K; kidney, Ov; ovary, Pl; placenta, Ut; uterine

On GD13, an inhalation mask was used to anesthetize the animals with a mixture of nitrous oxide/oxygen/isoflurane (69%/30%/1%) during surgical preparation. Body temperature was monitored and maintained at 37 °C. A 2 cm midline abdominal incision was performed to expose the uterine horns and their vascularization, live and dead fetuses were counted within the uterine horns, and surgery was performed. After trying many ligation places, it was found that ligation of uterine vessels in the middle of each uterine horn was suitable as described by Janot et al. [25]. Other ligation places resulted in either maternal death, maternal abortion, or complete fetal resorption. Briefly, the uterine vessels (artery and vein) were ligated (6–0 silk) at the central part of each uterine horn (Fig. 1a&b). Pregnant mice from sham-vehicle and sham-edaravone groups underwent sham procedure without ligation. After that, the uterus was placed back into the abdominal cavity, the abdominal wall was sutured, and the animals were housed individually and monitored. Maternal body weight was monitored from GD13 to GD18.

### Measuring the uterine blood flow

Blood flow (BF) values in both the right and left uterine arteries were obtained before and immediately after ligating uterine vessels. Blood flow was measured with a laser-Doppler flowmeter (FLO-C1, 97 Omegawave, Tokyo, Japan). A laser Doppler 98 flowmetry probe was fixed perpendicular to the uterine arteries where BF values were measured uninterrupted for five minutes. Three values were recorded at each artery. After ligation, BF values were rerecorded at the same places for another five minutes uninterrupted. The mean BF value was recorded and percentage of decreased BF after ligation was calculated.

### Experimental groups and treatment

The pregnant mice were divided into four groups (each *n* = 8), as follows:

1. Sham-vehicle (SV) group, sham procedure plus i.v. injection of 0.3 ml saline.
2. Sham-Edaravone (SE) group, sham procedure plus i.v. injection of 0.3 ml edaravone (Mitsubishi Tanabe Pharma, 3 mg/kg).
3. RUPP-vehicle (RV) group, surgery with ligation of both uterine vessels plus i.v. injection of 0.3 ml saline.
4. RUPP-Edaravone (RE) group, surgery with ligation of both uterine vessels plus i.v. injection of 0.3 ml edaravone (Mitsubishi Tanabe Pharma, 3 mg/kg) (Fig. 1c).

The edaravone dose was chosen after trying different doses; 3 mg/kg, 30 mg/kg and 100 mg/kg. It was found that the dose of 3 mg/kg had no adverse effects on the developing fetuses, however, the higher doses resulted in increased fetal loss. This dose was proven to be effective in many ischemic models [42]. All injections were done via the tail vein from GD 14 to GD 18. This study was based on 42 adults (32 females and 10 males) and 239 fetuses.

Blood pressure of conscious pregnant mothers was evaluated by recording the systolic blood pressure (SBP), diastolic blood pressure (DBP) and mean blood pressure (MBP) (mmHg) using a noninvasive tail-cuff method (BP-98A; Softron, Japan) [17].

### Maternal and fetal sampling

On GD 18, the pregnant females were decapitated under deep anesthesia by i.p. injection of pentobarbital (40 mg/kg), subjected to caesarian section, then the whole uterus with fetuses were removed and embryotoxicity was initially assessed by counting the number of live, resorbed and dead fetuses. Resorbed fetuses are those who are disintegrated and assimilated in the uterus after the completion of organogenesis. Dead or stillbirth fetuses were distinguished from live fetuses in terms of colour (normal pink vs. brown, green, black), movement and body consistency (normal, dry or soft). Live fetuses were anaesthetized by isoflurane and examined grossly for investigating the morphological abnormalities.

Fetal resorption and viability rates were calculated as the number of fetuses counted on GD13 minus the number of fetuses found on GD18 at the time of caesarian section. Fetal body weight, crown-rump length and placental weight were recorded in the four groups. Fetuses were then photographed and after that eviscerated and kept in 10% formalin for detection of endo-skeletal malformations.

### Biochemical, histopathological and immunohistochemical analyses

Maternal serum was obtained via centrifugation of maternal blood sample (from heart and ventral aorta) at 3500 r.p.m. at 4 °C for 10 min, the supernatant was collected and stored at − 80 °C [35]. For urea detection, a commercial urea assay kit (Abcam ab83362) was used according to manufacturer’s instructions. Serum creatinine was measured using creatinine assay kit (Abcam ab65340) according to manufacturer’s instructions. In both tests, colour was detected at OD of 570 nm.

For histopathology and immunohistochemistry, Maternal kidney and heart were fixed in 4% paraformaldehyde, cryoprotected in 30% sucrose, embedded and frozen at − 80 °C. Transverse renal or longitudinal cardiac frozen 20 μ thickness sections were cut and stored in − 80 °C. Hematoxylin and eosin staining was performed [21] and photographed using Olympus microscope (BX51).

Immunohistochemical procedure was performed according to Shang et al. [41], briefly, sections were incubated with rabbit anti-caspase3 (1:300, Cell Signaling Technology, Danvers, MA) primary antibody overnight at 4 °C, incubated with rabbit monoclonal biotinylated secondary antibody (1:200; Vector Laboratories, Burlingame, CA) for 2 h. at RT. Immunoreactivity was developed using horseradish peroxidase streptavidin-biotin complex solution (Vectastain ABC kit; Vector Laboratories) for 30 min. and then incubated with DAB.

### Endo-skeletal investigation of fetuses

For endo-skeletal preparation of fetus, double staining transparency technique was applied using the Alcain blue and Alzarin red S for staining cartilage and bone, respectively [3]. Photographs were taken and lengths of ossification centers within some bones were measured using Fiji image J software.

### Statistical analysis

All data sets were expressed as mean ± standard error of the mean (SEM). The data were analyzed statistically for normal distribution (student’s T test) and homogeneity of variances (Levene test) using statistical package of social sciences (IBM SPSS) statistics software for Windows, Version 22 (IBM Corp., Armonk, NY, USA). Differences were considered insignificant whenever *P* > 0.05. The significances of the obtained data were classified into two categories, i.e., *p* < 0.05 and *p* < 0.01 according to the obtained *p* values.

## Results

### RUPP surgery reduced the uterine artery blood flow

Ligation of uterine vessels in the middle of uterine horns at GD13 resulted in dramatic decrease in the arterial blood flow to mice fetuses with a percentage of 76.6% (from 54.7 ± 2.7 mm/s to 12.9 ± 1.7 mm/s, *p* < 0.01) (Fig. 1S).

### Body weight of mothers and uteri

**Fig. 2:**
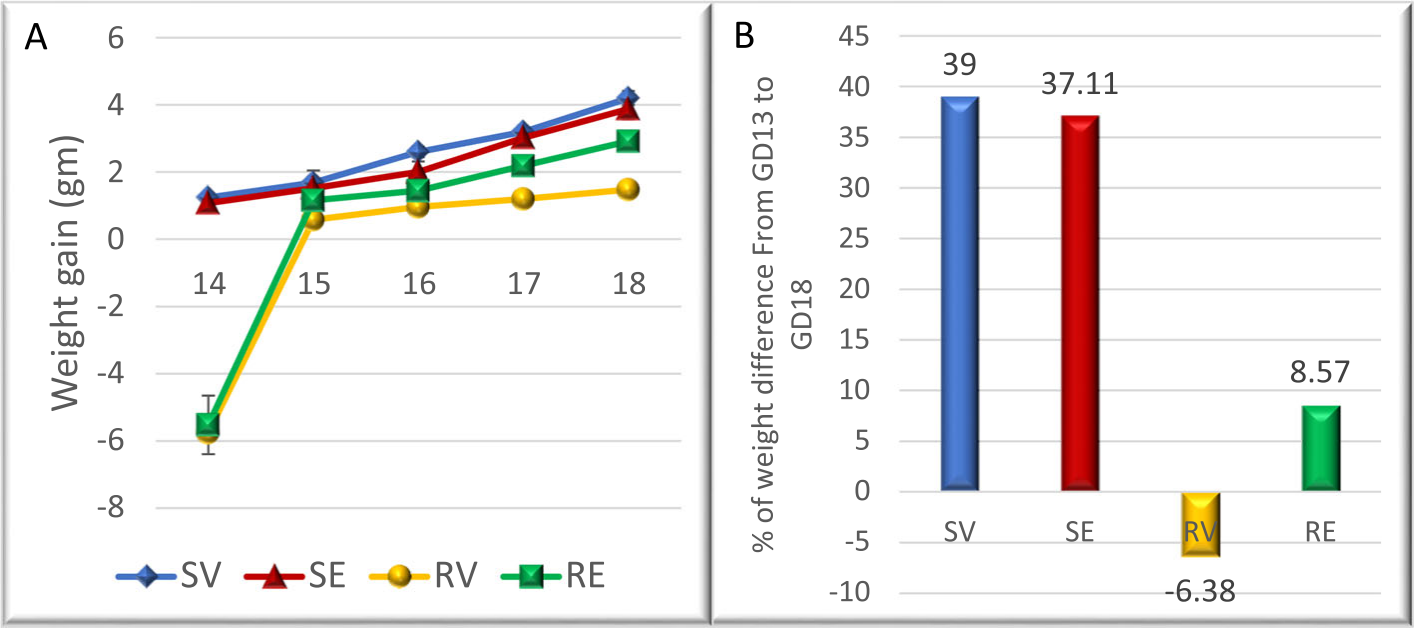
RUPP ischemia placental model decreases pregnant mother bodyweight gain and edaravone injection recovers body weight gain. **a** Differences of body weight gain of pregnant mice relative to GD 13 from different groups. **b** Percentage of total body weight gain of pregnant mice relative to GD13 body weight in different groups. RV mothers showed drastic decrease in their weight and couldn’t recover the initial weight decrease after surgery. RE pregnant mice showed better weight gain recovery, though still less than that seen in control

Mothers from either SV or SE groups showed continuous progressive increase in their weight (Fig. 2a, Table 1S) with a total weight gain percentage of 39 (SV) and 37.1 (SE) (Fig. 2b, Table 2S). On the other hand, pregnant mice from RV group showed drastic decline in their weight on the first day after surgery (− 5.8 ± 0.2 g), after that their body weight increased slowly in low values, however, they could not recover the initial body weight loss after surgery with a final percentage of body weight loss of − 6.4 (Fig. 2b, Table 2S). Contrarily, injection of edaravone to RUPP pregnant mice resulted in an increase in their body weight and recovery of the initial body weight loss, though still lower than the values of SV group (Fig. 2a, Table 1S) with a final net weight increase of 8.6 at the end of the experiment (Fig. 2b, Table 2S).

Regarding the uterine horns weight, there was insignificant difference between SV and SE groups. In contrast, pregnant mice from RV group showed a highly significant decrease in the uterus weight (*p* < 0.01) with almost 50% decline compared with SV group. Nevertheless, the uterine weight of RE mice increased significantly compared with RV group, though it showed significant decrease (*p* < 0.05) compared with SV group with a reduction percentage of 17.3 (Table 3S).

### Edaravone improves fetal growth and viability

**Fig. 3:**
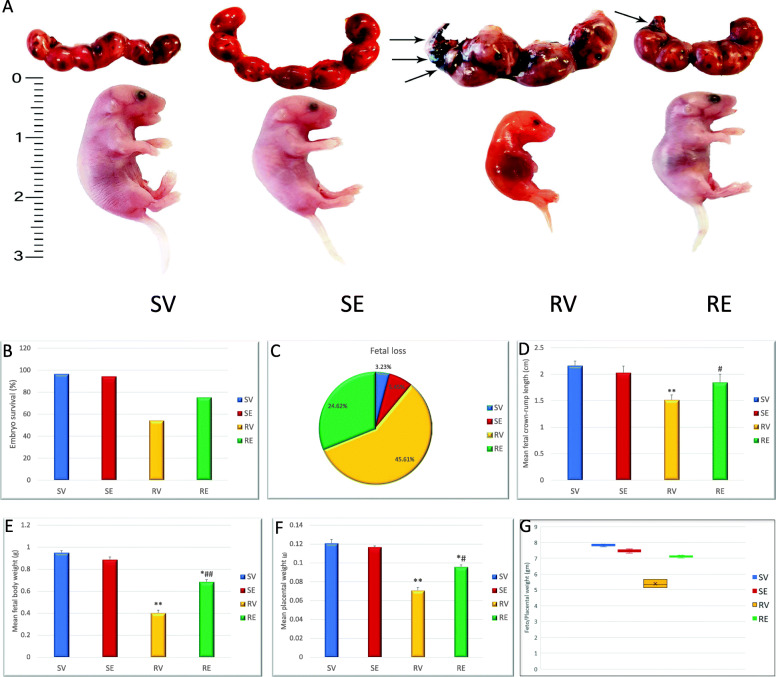
Pregnancy outcome of RUPP surgery and edaravone injection. **a** Representative photogrphs of uterine horns and fetuses from different groups showing fetal resorption (arrow) and growth retardation of fetuses from RV group. **b** Embryo survival showed its least value in RV group and increased after edaravone injection (RE) group. **c** Fetal loss is very high in RV group and decreased after edaravone injection. **d** Crown-rump length of fetuses from different groups at GD18. **e** Fetal weight from different groups showing highly significant decrease in RV group. **f** Placental weight from different groups. **g** Relationship between fetal to placental weight shows a highly significant decrease in RV group suggecting placental insufficiency due to RUPP surgery. This ratio increased after edaravone injection

The current study showed that RUPP placental ischemia model induced a dramatic decrease in fetal viability with increased percentage of fetal loss (45.6%) with more fetal resorption than fetal death. Interestingly, RE mice showed increased fetal viability compared with RV (75.4% (RE) and 54.4% (RV)). Both SV and SE showed high rate of fetal viability (96.8% (SV) and 94.6% (SE)) (Table 1, Fig. 3a-c).

**Table 1:** Embryotoxic and morphometric analysis of pregnancy outcome in the four different groups

	SV *n* = 62	SE *n* = 55	RV *n* = 57	RE *n* = 65
Mean number of fetuses at surgery (GD13)	8 ± 1	7 ± 1	7 ± 1	8 ± 1
Mean number of fetuses at sacrifice (GD18)	7 ± 1	6 ± 1	4 ± 1	6 ± 1
Fetal Loss	(2) 3.23%	(3) 5.45%	(26) 45.61%	(16) 24.62%
Fetal absorption	(2) 100%	(2) 66.67%	(18) 69.23%	(11) 68.75%
Fetal death	0%	(1) 33.33%	(8) 30.77%	(5) 31.25%
Fetal viability	96.77%	94.55%	54.39%	75.38%
Mean fetal crown-rump length (cm)	2.16 ± 0.081	2.03 ± 0.121	1.52 ± 0.089^**^	1.85 ± 0.151^#^
Mean fetal weight (g)	0.952 ± 0.017	0.888 ± 0.023	0.403 ± 0.025^**^	0.687 ± 0.017^*#^
Mean placental weight (g)	0.121 ± 0.004	0.117 ± 0.001	0.071 ± 0.003^**^	0. 096 ± 0.002^*#^
Mean fetal weight: placental weight	7.87	7.59	5.68	7.15

Data are represented as mean ± SD

* *p* < 0.05 ** *p* < 0.01 compared with the SV group

# *p* < 0.05 compared with RV group

RUPP surgery induced fetal growth retardation reflected by highly significant decrease (p < 0.01) in fetal body weight and crown rump length in RV group compared with SV group. Nevertheless, maternal injection of edaravone resulted in significant increase (p < 0.05) in fetal weight and crown-rump length versus RV group. There was no statistically significant difference between fetuses from SV and SE groups (Table 1, Fig. 3d&e).

The placental weight showed insignificant difference between SV and SE groups. Contrarily, there was a highly significant decrease in the placental weight in RV group which was significantly increased after injection with edaravone in the RE group. RV pregnant mice also showed placental insufficiency reflected by the decreased fetal to placental weight ratio with only 5.7 compared with the SV ratio of 7.9. It is noteworthy to mention that injection of edaravone also significantly increased the fetal to placental weight to reach 7.2 (Table 1, Fig. 3f&g).

### Edaravone decreases the fetal morphological abnormalities and improves fetus ossification

**Fig. 4:**
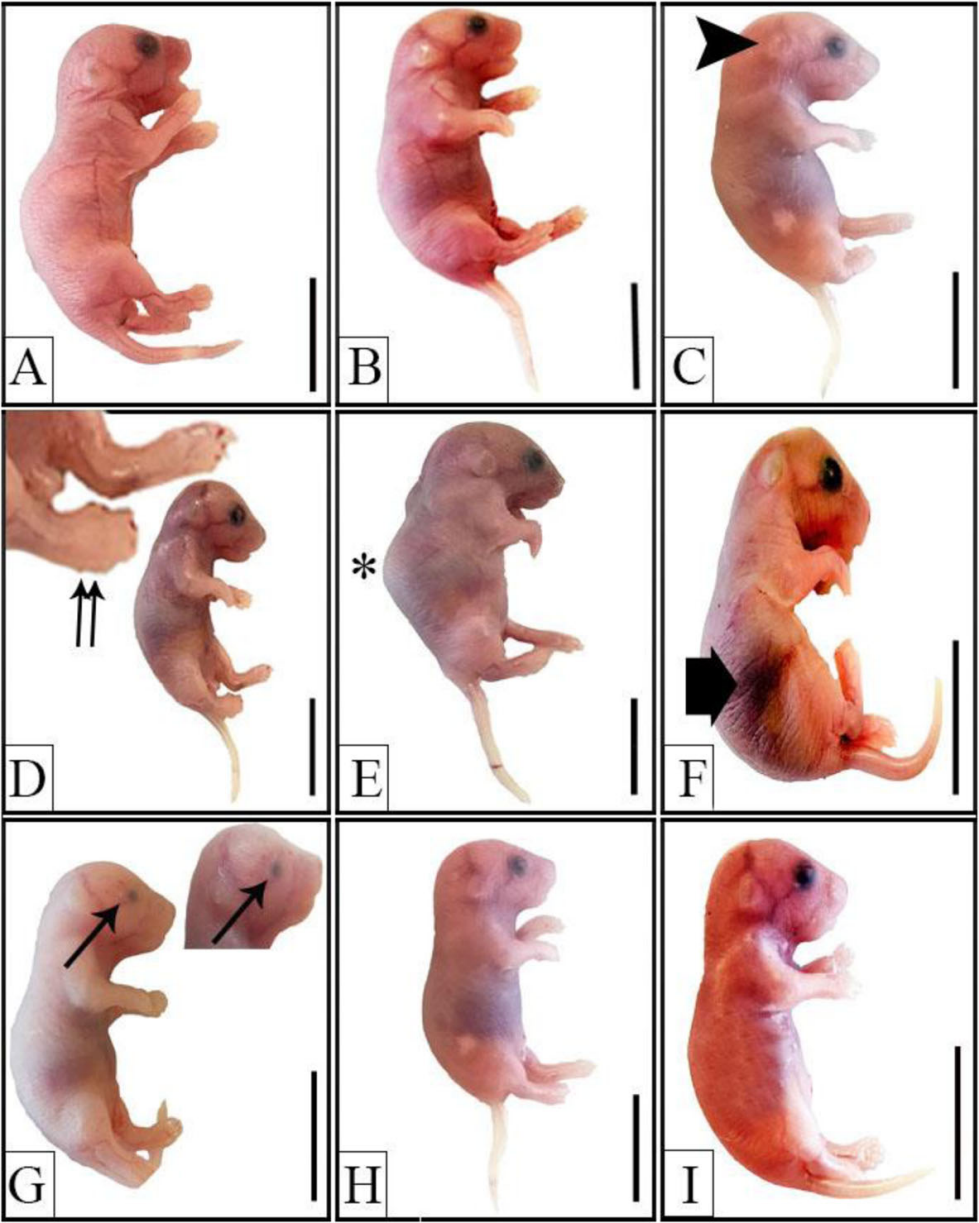
Representative photographs of macroscopic examination of GD18 fetuses from different groups. **a**&**b** normal morphology of fetuses from SV and SE groups, respectively. **c**-**h** fetuses from RV group showing various morphological anomalies including microtia (arrowhead), syndactyly (doubled arrow), kyphosis (asterisk), subcutaneous hemorrhage (thick arrow), unilateral microphthalmia (arrow) and small head (**h**). **i** fetus from RE group showing improved morphology after edaravone injection

GD18 fetuses from SV and SE groups showed normal morphological appearance (Fig. 4a&b). However, RV fetuses showed microtia (Fig. 4c), syndactyly (Fig. 4d), kyphosis (Fig. 4e), abdominal subcutanous hemorrhage (Fig. 4f), unilateral microphthalmia (Fig. 4g) and microcephaly (Fig. 4h). Fetuses from RE group showed more or less normal morphological structure (Fig. 4i).

**Fig. 5:**
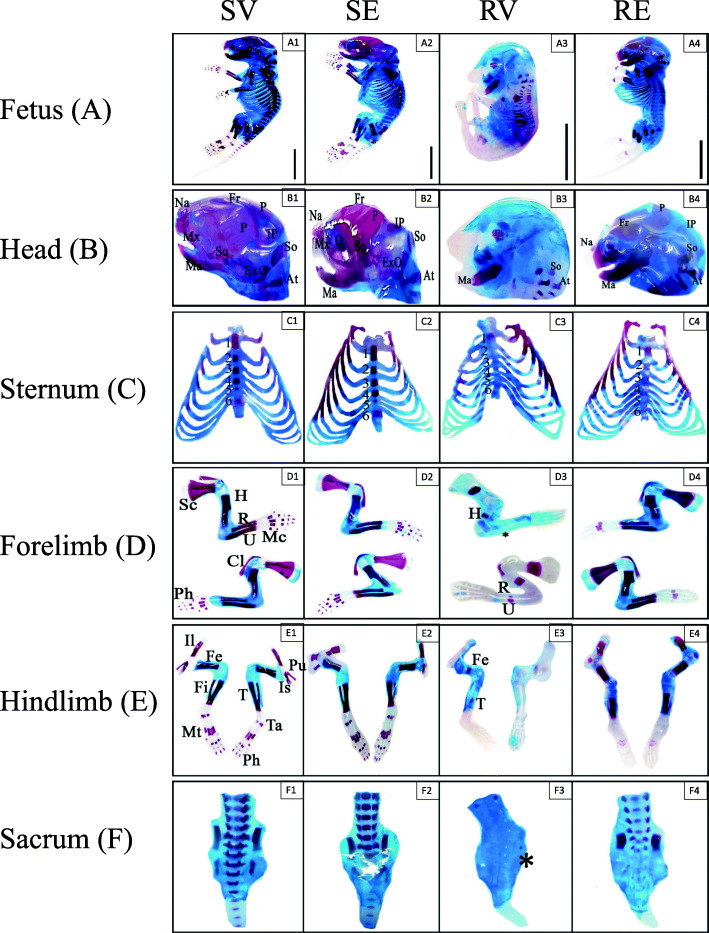
Representative photographs of double stained endoskeleton of fetuses and their body parts from different groups. (A) Whole fetus indicating severe loss of ossification in fetuses from RV group (A3). (B) Fetal skull showing well ossified bones in SV and SE groups (B1&2), while only supra-occipital and mandible were ossified in RV group (B3) and its amelioration after edaravone group (B4). (C) sternum and sternabrae ossification showing ossification of 6 segments in SV and SE groups (C1&2), 1st segment in RV group (C3) and first four segments in RE group (C4). (D&E) Well-ossified forelimb and pectoral girdle (D) and hindlimb and pelvic girdle (E) in SV and SE groups (1&2), severe delayed ossification in RV group (3) (asterisk) and amelioration after edaravone injection (4). (F) Ossification of lumbar and sacral vertebrae showing highly ossified centra in SV and SE groups (F1&2), complete loss of ossification in RV group (F3), except small portion in the ilium (asterisk), moderate amelioration after edaravone injection (F4). At, atlas; Cl, clavicle; ExO, exoccipital; Fr, frontal; Fe, femur; Fi, fibula; H, humerus; Il, Ilium; IP, interparietal; Is, ischium; Mc, metacarpus; Ma, mandible; Mt, metatarsus; Mx, maxilla; Na, nasal; O, orbit; P, parietal; Ph, phalanges; Pu, pubis; R, radius; Sc, scapula; So, supraoccipital; Sq, squamosal; Ta, tarsus; T, tibia; U, ulna

Endo-skeleton double staining showed that there was severe ossification loss in different body parts of RV fetuses, which was improved after edaravone injection (Fig. 5a). In the skull, only two bones were ossified compared with SV group which exhibited a large degree of ossification. This number increased after edaravone injection in RE group (Fig. 5b). Similarly, only the first sternabra was ossified in the sternum of RV fetuses, which increased to four after edaravone injection, while SV fetuses showed ossification of the whole six sternabrae (Fig. 5c).

RV fetuses displayed partial ossification in girdles, stylopodail and zeugopdial bones, however the autopodial bones didn’t show any ossification at all, in contrast to the case in SV group which showed high degree of ossification in all of the bones. This pattern increased after edaravone injection, although the autopod showed delayed ossification compared with SV group (Fig. 5d&e). The sacrum in RV fetuses remained completely cartilaginous unlike that of SV group. Edaravone injection partially restored the sacral ossification compared with RV group (Fig. 5f). SE fetuses showed similar ossification pattern with SV group.

There was no significant difference in the mean length of bone ossification centers from RV and SV fetuses. Conversely, RV fetuses showed highly significant decrease in ossification centers in all bones compared with SV group. Nevertheless, there was a highly significant increase in the ossification centers after edaravone injection compared with RV and significant decrease (*p* < 0.05) compared with SV group (Table 2, Fig. 2S).

**Table 2:** Effect of edaravone injection on the lengths (cm) of ossified centers of the long bones in GD18 mice fetuses subjected to RUPP placental ischemia

	SV	SE	RV	RE
Scapula	0.356 ± 0.018	0.342 ± 0.016	0.122 ± 0.008^**^	0.246 ± 0.023^*##^
Humerus	0.344 ± 0.009	0.326 ± 0.017	0.054 ± 0.009^**^	0.186 ± 0.011^*##^
Ulna	0.366 ± 0.015	0.348 ± 0.007	0.032 ± 0.008^**^	0.196 ± 0.014^*##^
Radius	0.292 ± 0.013	0.242 ± 0.017	0.020 ± 0.007^**^	0.158 ± 0.013^##^
Ilium	0.224 ± 0.011	0.202 ± 0.008	0.016 ± 0.005^**^	0.142 ± 0.010^##^
Ischium	0.148 ± 0.006	0.118 ± 0.008	0	0.038 ± 0.008^*##^
Femur	0.278 ± 0.008	0.248 ± 0.006	0.044 ± 0.013^**^	0.156 ± 0.016^*##^
Tibia	0.372 ± 0.009	0.351 ± 0.007	0.046 ± 0.011^**^	0.212 ± 0.016^*##^
Fibula	0.342 ± 0.008	0.316 ± 0.012	0	0.174 ± 0.015^*##^

Data are represented as mean (cm) ± SD

* *p* < 0.05 ** *p* < 0.01 compared with the SV group

## *p* < 0.01 compared with RV group. n = 5

### Edaravone improved maternal parameters and decreased the expression of cleaved caspase-3 in the maternal kidney

RUPP surgery induced hypertension in the mothers as it significantly increased SBP, DBP and MBP in RUPP vehicle versus sham vehicle groups. Edaravone injection significantly decreased these values, though still higher than that of the SV group. Meanwhile, there was no significant difference in all blood pressure parameters between SV and SE groups (Table 3, Fig. 3S A).

**Table 3:** systolic, diastolic and mean blood pressure (mmHg) of pregnant mice from different groups at GD 18

	SBP	DBP	MBP
SV	115.6 ± 3.162	79.2 ± 2.236	97.4 ± 2.573
SE	117.3 ± 2.915	80.7 ± 6.164	99 ± 2.850
RV	149.5 ± 4.123^**^	113.4 ± 6.016^**^	131.45 ± 1.917^**^
RE	128.2 ± 2.701^*##^	98.1 ± 4.929^*#^	113.15 ± 2.329^*##^

Data are represented as mean ± SD

* *p* < 0.05 ** *p* < 0.01 compared with the SV group

# *p* < 0.05 ## *p* < 0.01 compared with RV group. n = 5

Regarding kidney functions, RUPP significantly increased the levels of maternal serum urea and creatinine with an increase percentage of 52.8 and 88.9, respectively compared with SV group. Contrarily, there was a highly significant decrease in urea and creatinine concentration after edaravone injection compared with RV group with an increase percentage of 16.2 and 22.6 for urea and creatinine, respectively, compared with SV group. No significant difference was found between SE and SV groups (Table 4, Fig. 3S B).

**Table 4:** Serum levels of urea and creatinine of pregnant mice in different groups

	SV	SE	RV	RE
Urea conc. (mg/dl)	22.1 ± 0.13	23.4 ± 0.19	31.5 ± 0.15^**^	25.6 ± 0.14^*#^
Percentage of increase %	0	5.88%	42.5%	15.8%
Creatinine Conc. (mg/dl)	2.9 ± 0.11	3.1 ± 0.12	5.4 ± 0.14^**^	3.5 ± 0.11^*#^
Percentage of increase %	0	6.9%	86.2%	20.6%

Data are represented as mean ± SD

* *p* < 0.05 ** *p* < 0.01 compared with the SV group

# *p* < 0.05 ## *p* < 0.01 compared with RV group. n = 5

**Fig. 6:**
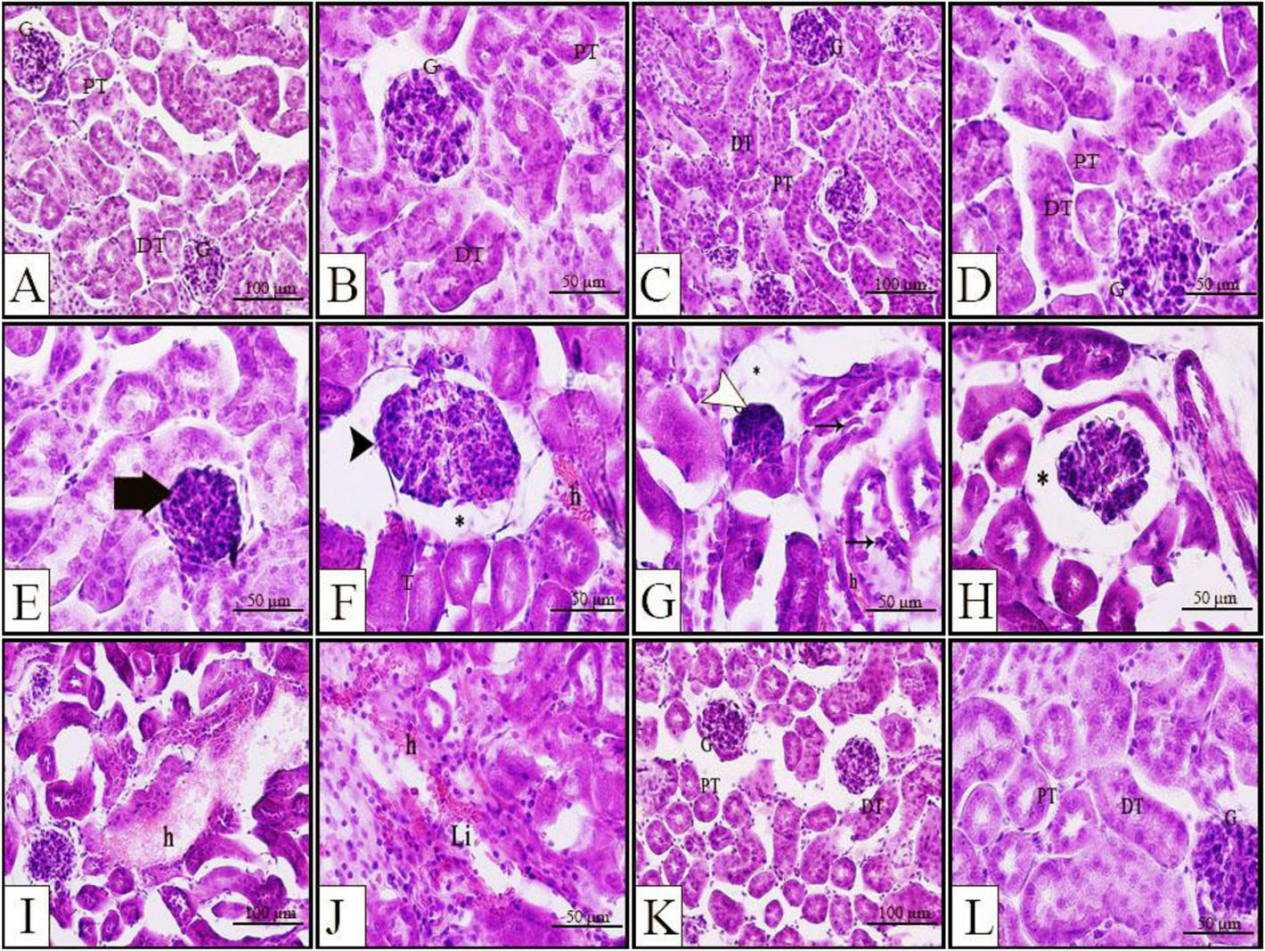
Representative photomicrographs of H&E stained transverse sections of maternal kidney from different groups. **a**&**b** SV group **c**&**d** SE group showing normal structure. **f**-**g** RV group showing endotheliosis (thick arrow), glomerulus swelling (black arrowhead), hemorrhage (**h**), shrunken glomerulus (white arrowhead), widened bowman’s space (*), and detached renal cells from degenerated tubules (arrow). **k**&**l** RE group showing improved renal structure after edaravone injection. DT; distal convoluted tubules, G; glomerulus, h; hemorrhage; Li; leukocytic infiltration, PT; proximal convoluted tubule

Histopathological examination of maternal kidney showed that both SV and SE groups had normal structure (Fig. 6a-d). On the other hand, adult kidney RV group showed capillary occlusion (Fig. 6e-j), endotheliosis (Fig. 6e), bleeding (Fig. 6f,g,i&j), occasionally aggregation of leukocytes (Fig. 6j), swelling of glomerulus with increase in cellularity (Fig. 6e&f) or shrinkage of glomerulus with widened bowman’s space (Fig. 6g&h), in addition to detached renal cells from degenerated tubules (Fig. 6g). However, all these alterations, except for capillary occlusion, were attenuated after edaravone injection in the RE group (Fig. 6k&l).

**Fig. 7:**
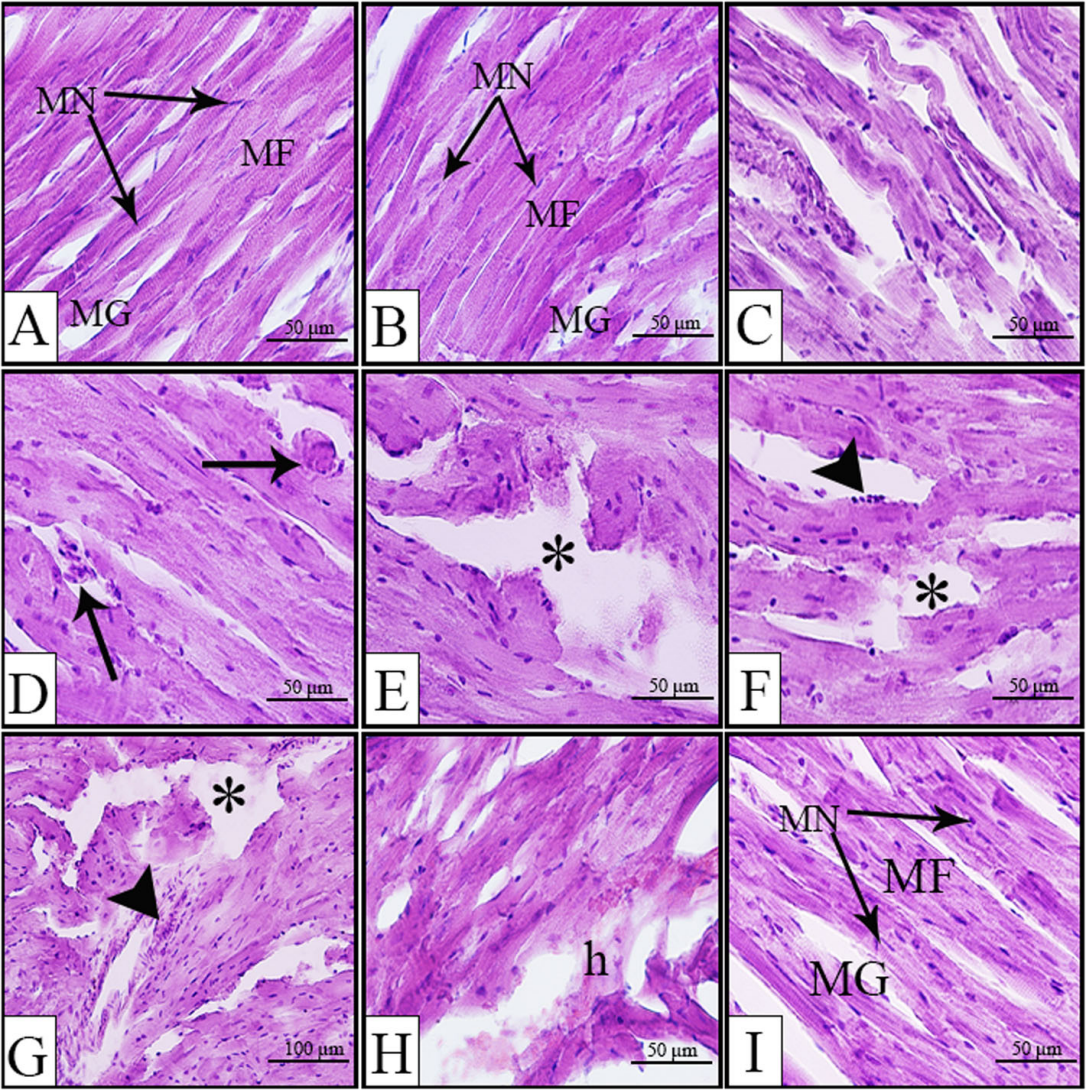
Representative photomicrographs of H&E stained longitudinal sections of maternal heart from different groups. Normal histological structure of heart from SV (**a**) and SE (**b**) groups. Maternal heart from RV group (**c**-**h**) showing irregular myocardial fibers (**c**), widened gaps between fibers with macrophage (arrow, **d**), necrotic foci (asterisk, **e**-**g**), cell mobilization from rippled muscle fibers (arrowhead, **f**&**g**), hemorrhage (h) within degenerated myofibrils (**h**). Improved heart structure after edaravone injection in the RE group (**i**). h; hemorrhage, MF; myocardial fibers, MG; myocardial gap, MN; myocardial nuclei

The maternal heart from both SV and SE groups showed well-arranged myocardial fibers (Fig. 7a&b for SV and SE groups, respectively). In contrast, the maternal heart from SV group showed focal myocyte necrosis (Fig. 7c-h), irregular myocardial fibers (Fig. 7c), wide myocardial gaps with macrophages (Fig. 7d), rippled myocytes with cell mobilization in the degenerating myocardial fibers (Fig. 7f&g) and hemorrhagic foci (Fig. 7h). Interestingly, edaravone injection resulted in improvement of the cardiac structure with minimal foci of damaged myocytes and a small irregularity in the muscle fibers (Fig. 7i).

**Fig. 8:**
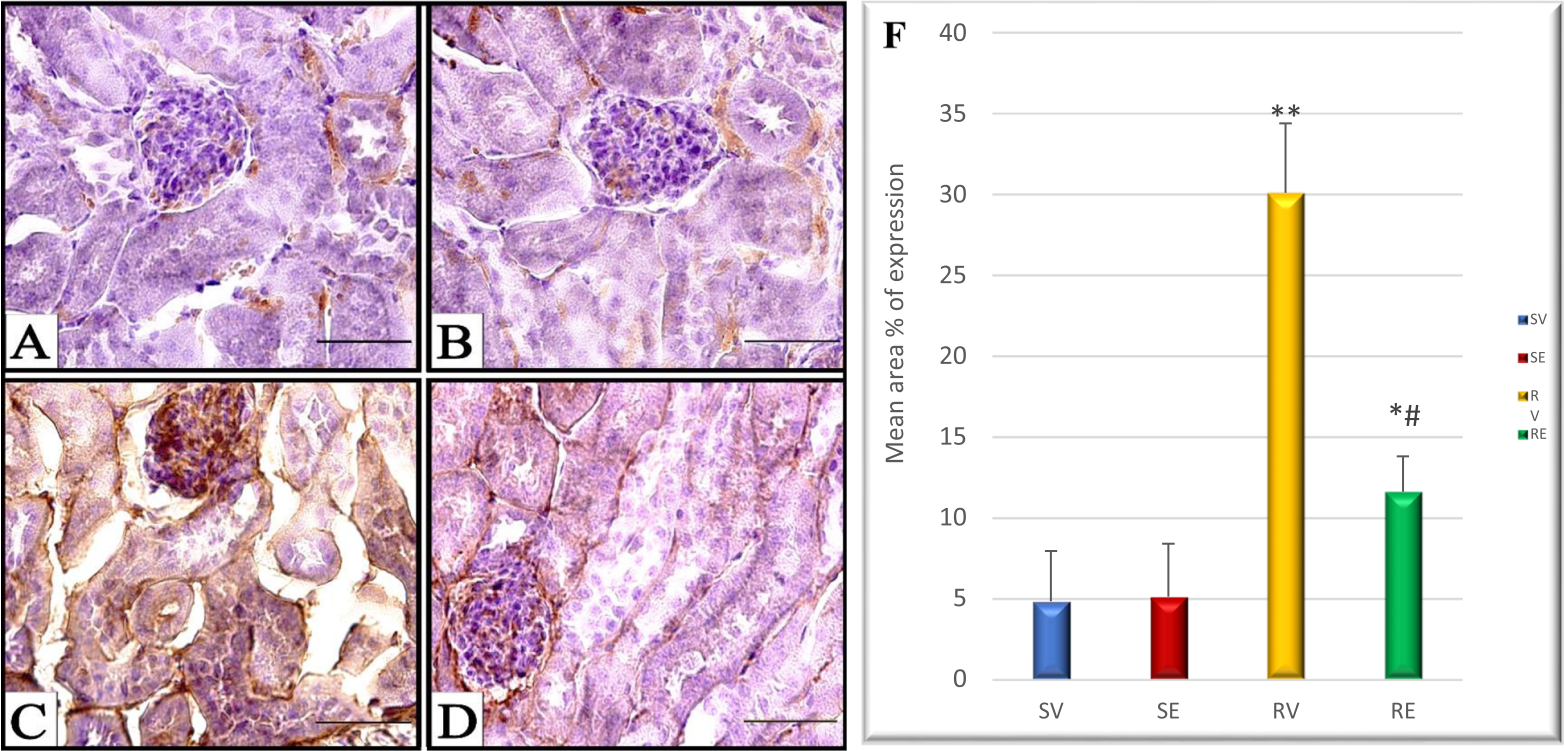
Photomicrographs showing immuno-histochemical localization of cleaved caspase-3 antigen in the maternal kidney. **a**&**b** SV and SE groups, respectively showing low cleaved caspase-3 expression. **c** RV group showing increased cleaved caspase-3 expression. **d** RE group with moderate expression. (Scale bar = 50 μm). **f** quantitative analysis of cleaved caspase-3 expression showing highly significant increase in the expression in RV group compared with SV group and highly significant decrease in the expression in RE group compared with RV group. # significant (*p* < 0.05) compared with RV group. *p < 0.05, **p < 0.01. *n* = 5 (number of sections from different dams). Data are represented as mean ± SD

Due to increased urea and creatinine levels in the blood suggesting malfunction of the maternal kidney, Caspase-3 immunohistochemical staining was adopted to investigate apoptosis. The expression of cleaved caspase-3 showed a high significant increase in the renal tubules and glomeruli of the kidney of pregnant mice subjected to RUPP with a mean percentage area expression of 30.1 compared with 4.8 in RV group. However, after edaravone injection the expression significantly decreased to 11.6%, though there was still significant increase compared with RV group. Meanwhile, there was insignificant difference between RV and RE groups (Fig. 8). The expression of cleaved caspase-3 in the maternal heart showed no significant difference among the four groups with low levels of expression (data not shown).

## Discussion

In the current study, RUPP induced severe reduction in pregnant mice weight which was accompanied by decreased uterine weights, increased fetal loss and resorption, as well as fetal growth retardation and decreased feto/placental ratio. Previous findings ascertained that RUPP induced maternal weight loss in different models [1, 17, 25, 50] accompanied by fetal growth retardation and decreased placental weight. According to Farag et al. [14], maternal and uterine weights decrease could be attributed to fetal intrauterine growth restriction and fetal loss. Fetal growth retardation is a hallmark of preeclampsia which is attributed to reduced placental blood flow to the growing fetus resulting in placental ischemia and as a consequence, reduction of maternal-fetal oxygen exchange and nutrients affecting fetal growth and development [19, 54].

Edaravone injection (3 mg/kg), in the current study, significantly increased the body weight gain of the mothers, significantly increased the fetal crown-rump length, weight, placental weight and feto/placental ratio. Edaravone is an established free radical scavenger which has a hydroxyl radical quenching effect and an inhibitory effect against peroxynitrite and peroxyl radicals [53]. One of the important factors leading to fetal growth restriction in preeclampsia is the increased production of placental oxidants due to increased maternal and fetal metabolism [10, 48]. The role of antioxidants in management of placental ischemia-induced growth retardation and decreased placental weight has been investigated [36, 43, 52]. Moreover, Ushida et al. [50] found that maternal administration of the novel antioxidant compound H_2_ to RUPP rats increased the fetal and placental weights. H_2_, same as edaravone, selectively reduces peroxynitirite, which has a pathological role on preeclampsia [29].

Mice fetuses of RUPP group in the current study showed many morphological abnormalities which could be attributed to the induced placental hypoxia according to Ritchie et al. [38] who reported that hypoxia induced by clamping of uterine arteries resulted in morphological malformations. Meanwhile, the fetuses of RUPP group, in the current study, showed severe delayed ossification and decreased length of ossified centers in different bones. Many studies had related disrupted bone development and skeletal abnormalities with fetal growth restriction [6, 39]. Additionally, hypoxia may play role in altered bone development [9, 51].

Interestingly, edaravone injection, in the present study, resulted in better morphological features of the fetuses, increased the fetal weight and length and increased the degree of ossification and length of ossified centers, though some skeletal structures showed delayed ossification compared with sham group. The cytoprotective effect of edaravone on bone and osteoblast survival through its ROS-scavenging ability has been reported in many studies [8, 46], moreover, the authors suggested that edaravone may have antiosteoporosis potential.

The present study showed that RUPP resulted in maternal hypertension, in addition, the maternal heart manifested many histopathological alterations. Maternal hypertension and cardiac abnormalities have been associated with RUPP procedure [17, 18, 23, 27], which can be attributed to placental oxidative damage and angiogenic imbalance [50]. Preeclampsia was reported to be associated with cardiac dysfunction and increased risk of myocardial infarction as well as heart failure [4, 31]. Additionally, hypertension, itself, can greatly increase the risk of cardiovascular diseases [7].

Edaravone injection, in the current study, attenuated that increase in blood pressure and the cardiac structure. Many studies have addressed the effectiveness of edaravone as a cardio-protectant, especially in ischemia/reperfusion injury [33, 45, 55], possibly via its hydroxyl radicals quenching properties [12] and its anti-inflammatory effect [26]. The maternal blood urea and creatinine levels were significantly increased in this study, this was accompanied by renal histopathological alterations in the RUPP mothers. Similar results showed that RUPP in pregnant mice resulted in increased albumin/creatinine ratio, endotheliosis, bleeding, glomerular swelling and mesangial expansion [17, 50]. Glomerular endotheliosis is usually a characteristic feature of preeclampsia [44]. It was suggested that increased oxidative stress can have deleterious effects on renal function in the RUPP model [5, 40]. In line with that, edaravone injection restored the renal functions and attenuated the renal structure. Edaravone was reported to ameliorate the renal ischemia/reperfusion injury and decreasing blood urea and creatinine possibly by scavenging the free radicals in the renal tubes and inhibition of lipid peroxidation [13]. In addition, edaravone alleviated the RUPP induced apoptosis in the maternal kidney as it decreased the expression of cleaved caspase-3. Previous reports showed the anti-apoptotic effect of edaravone and decreased caspase-3 expression in different ischemia models and attributed this effect to its free radical and ROS scavenging abilities [2, 16, 57].

In conclusion, the present study demonstrated that the RUPP-induced placental ischemia in the mice resulted in preeclampsia-related symptoms. Notably, edaravone injection attenuated most of these symptoms possibly due to its antioxidant and free radical scavenging ability. These results suggest the potential efficacy of edaravone as an adjuvant therapy for preeclampsia. However, further studies are needed to understand its actual mechanism of action during preeclampsia, as well as its safety for clinical use, to validate its application in the future.

## Supplementary Information

**Additional file 1.**

## Data Availability

The data that support the findings of this study are available within the article and its supplementary material.
