# Supplementary material for: Effect of edaravone on pregnant mice and their developing fetuses subjected to placental ischemia
Source: Reprod Biol Endocrinol. 2021 Feb 6;19:19. doi: 10.1186/s12958-021-00707-2 (PMC7866881; doi:10.1186/s12958-021-00707-2)
Supplement: Supplementary file 1 — Additional file 1. [file 12958_2021_707_MOESM1_ESM.docx]

Supplementary data


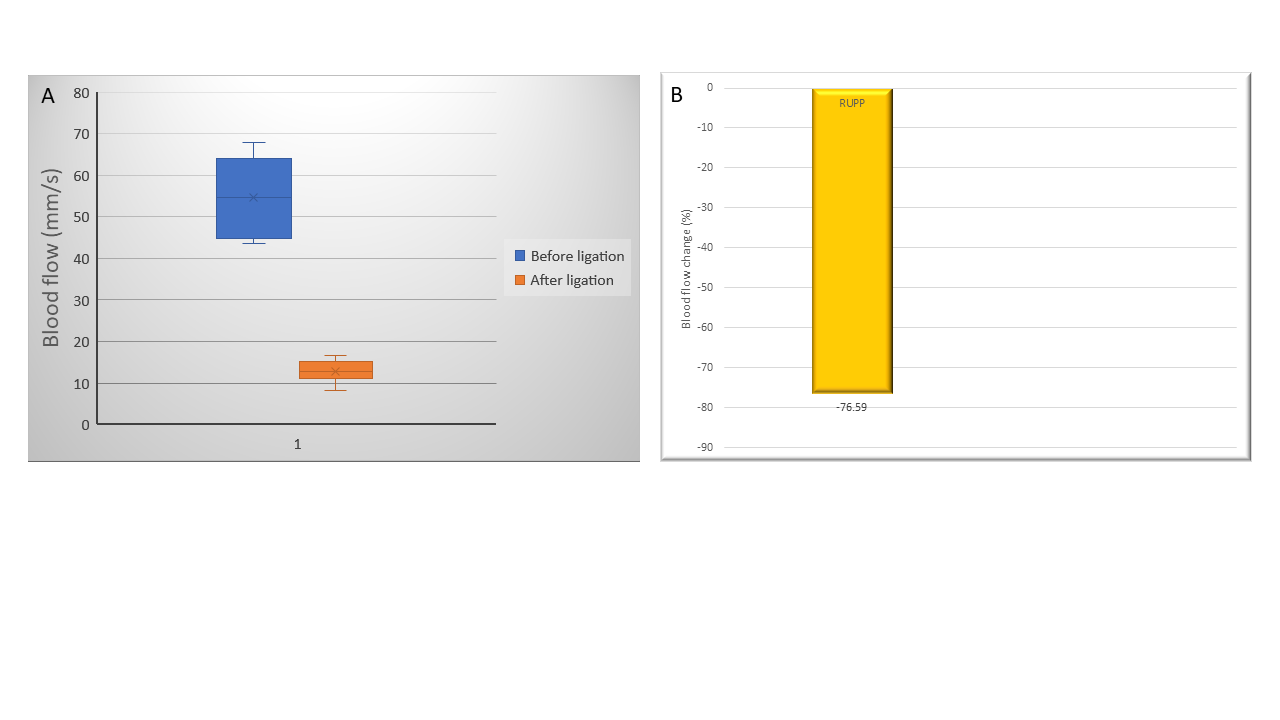


Fig. 1S: **Changes of uterine artery blood flow before and after RUPP surgery**. (A) the blood flow decreased dramatically after ligation of uterine vessels. (B) percentage of blood flow change after RUPP surgery relative to the flowrate before surgery.

Fig. 2S: Graphs showing the difference in the mean length of ossification centers in scapula and forelimb bones (A) and pelvic girdle and hindlimb (B) in different groups.

Fig. 3S: Graphs showing effect of RUPP on blood pressure at GD18 (A) and kidney functions (B) indicated by serum urea and creatinine levels of pregnant mice and its amelioration by edaravone.


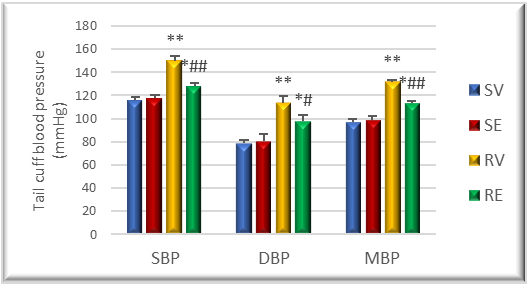


A


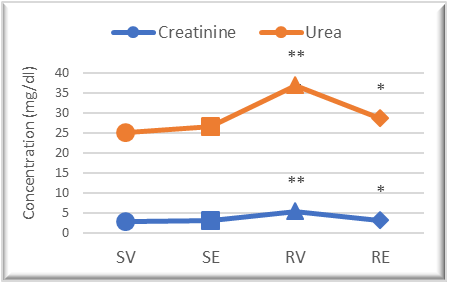


B

**Table (1S): Changes in the body weight gain of the mothers in different groups.**

| Group | Gestation day | | | | |
| --- | --- | --- | --- | --- | --- |
|  | 14 | 15 | 16 | 17 | 18 |
| SV | 1.24±0.114 | 1.68±0.363 | 2.6±0.158 | 3.2±0.114 | 4.2±0.212 |
| SE | 1.08±0.178 | 1.52±0.286 | 2±0.316 | 3.02±0.164 | 3.88±0.311 |
| RV | -5.76±0.236 | 0.58±0.216 | 0.96±0.181 | 1.2±0.158 | 1.48±0.178 |
| RE | -5.52±0.87 | 1.16±0.151 | 1.45±0.207 | 2.19±0.158 | 2.92±0.258 |

**Data are represented as mean ± SD.**

**Table (2S): Mean body weight, total weight gain and percentage of weight gain of pregnant mice from GD13 to GD18 in different groups.**

|  | SV | SE | RV | RE |
| --- | --- | --- | --- | --- |
| GD13 | 33.6 | 29.1 | 28.2 | 28 |
| GD18 | 46.7 | 39.9 | 26.4 | 30.4 |
| Total weight gain (g) | 13.1 | 10.8 | -1.8 | 2.4 |
| % of total weight gain | 39 | 37.11 | -6.38 | 8.57 |

**Data are represented as mean ± SD.**

**Table (3S): Weight of uteri of pregnant mice at GD 18.**

|  | Average weight of uteri | C% |
| --- | --- | --- |
| SV | 7.11±0.201 | 0 (0%) |
| SE | 6.95±0.172 | -0.16 (-2.25%) |
| RV | 3.53±0.174^**^ | -3.58 (-50.35%) |
| RE | 5.88±0.382^*##^ | -1.23 (-17.29) |

C% = percentage of change compared with control.

* p<0.05 ** p<0.01 compared with the SV group

## p<0.01 compared with RV group. n=8 per group.
